# Anti-infectives Developed as Racemic Drugs in the 21st Century: Norm or Exception?

**DOI:** 10.1021/acsmedchemlett.3c00214

**Published:** 2023-06-12

**Authors:** Diego González Cabrera, Dennis A. Smith, Gregory S. Basarab, James Duffy, Thomas Spangenberg, Kelly Chibale

**Affiliations:** † Drug Discovery and Development Centre (H3D), University of Cape Town, Rondebosch 7701, South Africa; ‡ Independent, 4 The Maltings, Walmer, Kent CT147AR, United Kingdom; § Medicines for Malaria Venture, Geneva 1215, Switzerland; ∥ Global Health Institute of Merck, Ares Trading S.A., Route de Crassier 1, 1262 Eysins, Switzerland; ^⊥^South African Medical Research Council Drug Discovery and Development Research Unit, Department of Chemistry, and ^#^Institute of Infectious Disease and Molecular Medicine, University of Cape Town, Rondebosch 7701, South Africa

**Keywords:** Anti-infective, Racemate, Single enantiomer, Chiral switch, New chemical entity (NCE)

## Abstract

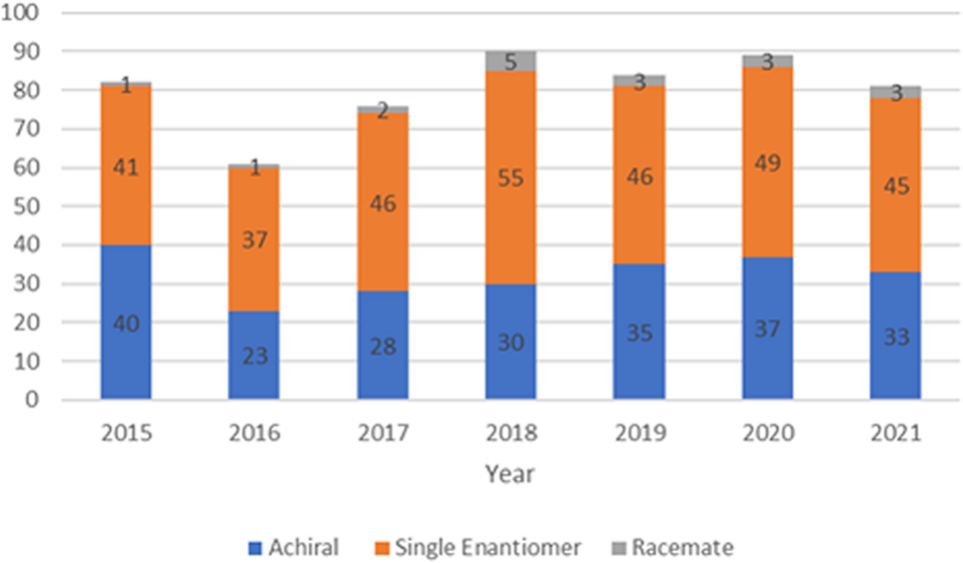

This viewpoint outlines the case for developing new chemical entities (NCEs) as racemates in infectious diseases and where both enantiomers and racemate retain similar on- and off-target activities as well as similar PK profiles. There are not major regulatory impediments for the development of a racemic drug, and minimizing the manufacturing costs becomes a particularly important objective when bringing an anti-infective therapeutic to the marketplace in the endemic settings of infectious diseases.

Developing single-enantiomer drugs has become the standard practice across the pharmaceutical environment. The practice is deeply embedded and is generally not subject to questioning in many organizations. Part of the standardization on single-enantiomer drugs is the belief that they are intrinsically superior in all aspects to the drug as a mixture of enantiomers. This paper examines the notion of superiority in terms of efficacy, safety, and regulatory acceptance, with the view that an evidence-based approach should be used to guide the decision to develop a single enantiomer or a racemate. It is worth noting that the potential technical challenges associated with manufacturing a single-enantiomer drug can result in increased cost of goods (CoG). With many therapeutic areas, CoG is not considered a major impediment; however, with new anti-infectives targeted against such diseases as malaria, the CoG and resultant cost of a treatment course is a significant issue for both patients and health care providers in the areas where the disease is endemic.

## Racemic Drugs as the Norm

Optical isomerism of organic molecules and their different affinities to chiral biological targets have been known for more than a century.^1,2^ In 1848, Louis Pasteur discovered that crystals of the sodium ammonium salt forms of tartaric acid existed in two mirror-image forms that rotated the plane of polarized light in opposite directions. He later noted that these salts were fermented differently by yeast and molds.^3^ However, it was not until 1874 that van’t Hoff and Le Bel independently explained the relationship between optical activity and molecular structure,^4^ laying the foundation of modern stereochemistry.^5^ A few decades later, Cushny described that one of the enantiomers of hyoscyamine displayed higher activity in the periphery of the nervous system while the other was more potent against the central nervous system, thus demonstrating that the pharmacological properties of single enantiomers could differ from each other.^6,7^

Despite this knowledge, the majority of synthetic chiral pharmaceuticals were administered as racemates rather than single-enantiomer products throughout most of the 20th century.^1,2^ This has been a consequence of the difficulty and expense of commercial chiral separation of racemates and/or of production of single enantiomers on a large scale.^1,2,8^

## The Move to Single Enantiomer as the Dominant Form

In the 1980s and 1990s, advances in chiral separation technologies and asymmetric synthesis, together with a growing recognition that enantiomers may have distinct pharmacodynamic (PD) and/or pharmacokinetic (PK) profiles,^9−12^ impacted the drug design and drug discovery strategies and policies of the pharmaceutical industry, creating a new focus on the development of achiral or single-enantiomer drugs as preferred entities.^13^ Due to this increased awareness of the implications of enantiomers in determining therapeutic outcomes, both the United States (U.S.) Food and Drug Administration (FDA) and the European Union (EU) Committee on Proprietary Medicinal Products (CPMP) issued formal guidelines for the development of new stereoisomeric drugs in the early 1990s.^3^

The view that single-enantiomer drugs could show superior therapeutic outcomes to racemates is based on five assumptions.^3^

1. The dose could be lowered through elimination of an inactive component.
2. Patient PK could be improved, and interpatient variability could be lowered.
3. Patient PD could be adjusted, and interpatient variability could be lowered.
4. The dose–response relationship could be simplified.
5. Toxicity could be minimized or eliminated.

Retrospective analysis of the experience with racemic therapeutics has suggested that these assumptions are overly simplistic.^3^ In some cases, single-enantiomer chiral drugs outperform the corresponding racemates, typically when the pharmacological effect resides largely in one of the enantiomers. For example, the (S)-enantiomer of bupivacaine is significantly less cardiotoxic than the racemate and the (R)-enantiomer.^13^ In other cases, especially in the field of infectious diseases, and more specifically in malaria, racemic drugs (e.g., chloroquine, tafenoquine, lumefantrine, and other related compounds) are administered to treat the disease.^3^

The trend to choose single-enantiomer drugs over racemates has sometimes been driven by factors that are distinct from pharmacology: (i) the business decision to extend product life by replacing an existing racemic drug to one of the enantiomers (“chiral switch”)^13−15^ or (ii) the *de novo* selection of a single enantiomer in the belief that this will simplify the development pathway.^13^ In the environment of infectious disease drug development, where cost pressures are significant due, in part, to considerable poverty in the endemic settings, the generally increased costs for developing and manufacturing single-enantiomer agents may limit access to novel medicines.

Although the first two drugs, dilevalol and dexfenfluramine, to be approved as single enantiomers from clinically established racemates were subsequently withdrawn, chiral switching has been an important practice in drug development since 1994.^14,15^ However, chiral switching has sometimes proved controversial, especially when the single-enantiomer form does not provide improved efficacy or safety over the racemate, but simply allows manufacturers to extend patent protection against generic competitors.^13,16,17^ Furthermore, the validity of a number of patents on single-enantiomer drugs obtained via a chiral switching strategy have subsequently been challenged under Paragraph IV certification, which incentivizes the entry of generic drugs into the market in various patent jurisdictions.^16^

A recent analysis^17^ suggests that oftentimes the chiral switching strategy, while successful for the purpose of creating new intellectual property, often fails to produce a superior agent. A total of 15 drug pairs (enantiomer/racemate) were examined; the authors noted that, despite the argument for superiority, relatively few studies directly compare the new single enantiomer with the original racemic drug. Of 174 randomized-controlled studies that allowed for comparison based on efficacy, 83% showed no discrimination of the single-enantiomer drug versus the racemate at the level of the primary end point, 13% favored the single-enantiomer version of the drug, while 3% favored the racemic version. A subset of these studies also allowed for a comparative assessment of safety between the two agents. Within this subset, 86% showed no benefit of choosing the single enantiomer. Although the results above do not predict the fate of a future racemic development candidate, they do suggest that concerns about developing racemates have historically been overstated.^17^

## Changes to the Chemistry Process

There are three main pathways for *de novo* development of enantiomerically pure drugs:^13^

1. Using chiral starting materials.
2. Applying asymmetric synthesis strategies.
3. Employing chiral separation of a racemic intermediate or product.

During the early phases of a drug discovery program, it is often time- and cost-effective for an organization to perform structure–activity relationship (SAR) studies using racemic forms of chiral compounds. Many relevant properties can be successfully evaluated using racemates, which are often easier and less costly to prepare. Once a small subset of optimized drug candidates has been identified, separation and evaluation of the individual enantiomeric partners in a racemic pair becomes a crucial step. The results of this analysis can guide a decision to choose between the original racemate and a preferred enantiomer. If the pharmaceutical company decides to focus on one of the two enantiomers, asymmetric synthesis to supply greater amounts of the enantiomerically pure sample becomes a critical-path activity. Large quantities are needed for preclinical research, development, and clinical trials before regulatory authorities’ approval of the single-enantiomer drug.^13^ If the racemate remains the preferred agent, additional data (see FDA–EMA Guidance below) to support an eventual filing will need to be collected on the individual enantiomers.

## The Current View on Racemic Drugs

**Figure 1:**
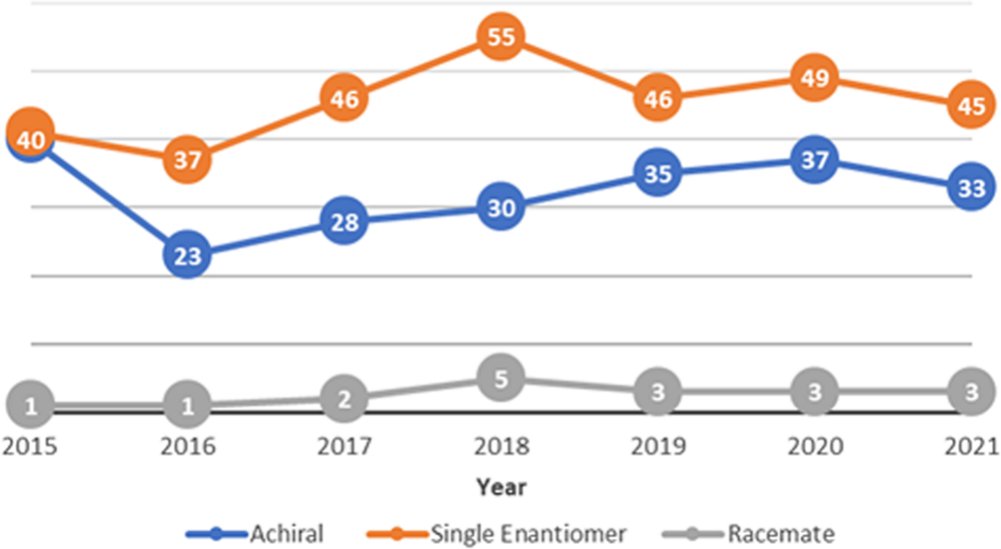
Number of NCEs, approved or entering phase III, by year.^18^

The pharmaceutical industry has responded to the challenge of chirality through different strategies. Achiral compounds can often be assembled in a straightforward manner from readily available commercial starting materials; as such, it is considered desirable to identify achiral drug candidates, and nearly 40% of all NCEs identified during the past decade lack chirality. The majority of chiral NCEs have been developed as single enantiomers; however, racemic drugs persist in the drug development pipeline, comprising ∼5% of the chiral pool (see Figure 1).

In the field of infectious diseases, and more specifically in the area of antimalarial drugs,^3^ tafenoquine, chloroquine, hydroxychloroquine, quinacrine, primaquine, mefloquine, halofantrine, and lumefantrine are all chiral molecules that are administered as racemates, suggesting that these are the norm rather than the exception.^19^ Minimizing the CoG becomes a particularly important goal when bringing anti-infective therapeutics to the marketplace in an endemic setting. Parallel profiling of both a potential racemic drug and its respective enantiomers (including CoG analysis) should be a focus during preclinical discovery and development phases. In the case where both enantiomers retain similar on- and off-target activities as well as similar PK profiles, the decision to exclude one becomes difficult to justify. Of note, the physicochemical properties of a mixture of enantiomers may differ from those of the pure enantiomers in ways that are relevant to the development pathway. For example, it is likely that the crystal species will be different in ways that are either beneficial or detrimental. Whether a mixture of enantiomers or a single enantiomer forms crystals of higher thermodynamic stability varies on the chemical entity.^20^ Lower thermodynamic stability leads to higher thermodynamic and kinetic solubilities, which would be an asset if drug solubility was limiting. Conversely, higher thermodynamic stability can result in a longer shelf life of both the active pharmaceutical ingredient (API) and the drug product/formulation for clinical use. Shelf life is another important parameter for anti-infective drugs due to storage limitations (e.g., if cold temperatures are required) and high humidity in many endemic settings.

Thus, development should proceed forward with the racemate until and unless some relevant differentiated property is identified. *In vitro* and *in vivo* studies can be performed with separated enantiomers; *in vivo* PK studies using the racemate (monitored with chiral analytics) can also provide data on the individual enantiomers.^21,22^

## Regulatory Environment and Development Status

When choosing between a racemic drug and its individual enantiomers, the complexity of the development path is clearly one factor. As a homogeneous single entity, the regulatory and development path for an enantiomeric drug is now clearly established. What additional complications, if any, are introduced when pursuing a mixture of enantiomers?

From stringent regulatory authorities’ perspective, the FDA and European Medicine Agency (EMA) have offered similar guidance on whether and how to prosecute a development plan for a racemic drug. The FDA Guidance Document, “Development of New Stereoisomeric Drugs”,^23^ was first created and released in 1992 but has been maintained without alteration through multiple reviews and is current as of 2018. The latest EMA guidance, “Investigation of Chiral Active Substances”, was released in 1994.^24^ The FDA document notes that advances in asymmetric synthesis and chiral chromatographic purification have brought the question of developing stereoisomers to the forefront. It makes clear that diastereomers (where the relative orientation of substituents at the two or more stereogenic centers is different between isomers) “should, with the rare exception of cases where *in vivo* interconversion occurs, be treated as separate drugs and developed accordingly”,^23^ but it distinguishes these from enantiomers. The FDA is agnostic on the question of developing racemates versus enantiomers but rather provides guidance to justify the choice of one form or the other.

## FDA–EMA Guidance

- Quantitative assays for the individual enantiomers need to be developed to allow assessment of the potential for interconversion and the absorption, distribution, biotransformation, and excretion (ADBE) profile of the individual isomers. If it is established that the pharmacokinetic profile is similar for both, an achiral assay should suffice for later evaluation.
- When possible, the activity of individual enantiomers should be established in *in vitro* systems, in animals and/or in humans.
- A relatively benign toxicologic profile using the racemate would ordinarily support further development without separate toxicologic evaluation of the individual enantiomers. However, any unusual or low-therapeutic index toxicology findings should be evaluated with the individual enantiomers.^23,24^

Overall, the FDA Guidance notes that “the common practice of developing racemates has resulted in few recognized adverse consequences,” The observation of additivity or synergy between enantiomers in a racemic pair is also noted as a potential advantage and would apply to areas such as potentially limiting the emergence of drug resistance.

As it appears that there are not major regulatory impediments to the development of a racemic drug, the only additional requirements of note are the preclinical characterization of both enantiomers *in vitro* and *in vivo*, plus the development of a quantitative assay to assess the enantiomers in biological samples. If no unanticipated signals are observed during *in vitro* toxicology studies, *in vivo* toxicology studies can be performed on the racemate only, and thus, further development should proceed forward with the racemate until and unless some relevant differentiated property is identified.

## Abbreviations

CoG: cost of goods
NCE: new chemical entity
PD: pharmacodynamic
PK: pharmacokinetic
FDA: U.S. Food and Drug Administration
CPMP: Committee on Proprietary Medicinal Products
SAR: structure–activity relationship
API: active pharmaceutical ingredient
EMA: European Medicine Agency
ADBE: absorption, distribution, biotransformation, and excretion
